# Congenital microcephaly: Case definition & guidelines for data collection, analysis, and presentation of safety data after maternal immunisation

**DOI:** 10.1016/j.vaccine.2017.01.044

**Published:** 2017-12-04

**Authors:** Malini DeSilva, Flor M. Munoz, Erick Sell, Helen Marshall, Alison Tse Kawai, Alisa Kachikis, Paul Heath, Nicola P. Klein, James M. Oleske, Fyezah Jehan, Hans Spiegel, Mirjana Nesin, Beckie N. Tagbo, Anju Shrestha, Clare L. Cutland, Linda O. Eckert, Sonali Kochhar, Azucena Bardají, for The Brighton Collaboration Congenital Microcephaly Working Group

**Affiliations:** a Health Partners Institute for Education and Research, United States; b Baylor College of Medicine, United States; c Children’s Hospital of Eastern Ontario, Canada; d Vaccinology and Immunology Research Trials Unit, Women's and Children's Health Network and Robinson Research Institute and School of Medicine, University of Adelaide, South Adelaide, Australia; e Department of Population Medicine, Harvard Medical School & Harvard Pilgrim Health, United States; f Department of Obstetrics and Gynecology, University of Washington, School of Medicine, Seattle, WA, United States; g St. Georges Vaccine Institute, Institute of Infection & Immunity, St. Georges University of London, London, UK; h Kaiser Permanente Vaccine Study Centre, Oakland, CA, United States; i Rutgers New Jersey Medical School, Newark, NJ, United States; j Department of Paediatrics and Child Health, Aga Khan University, Pakistan; k Kelly Government Solutions (KGS), Contractor to DAIDS/NIAID/NIH, Rockville, United States; l National Institutes of Health/National Institute of Allergy and Infectious Disease, United States; m Institute of Child Health & Department of Paediatrics, University of Nigeria Teaching Hospital, Enugu, Nigeria; n Sanofi Pasteur, Global Pharmacovigilance, Sanofi Pasteur, United States; o Medical Research Council: Respiratory and Meningeal Pathogens Research Unit, Johannesburg, Department of Science and Technology National Research Foundation, Vaccine Preventable Diseases, Faculty of Health Sciences, University of the Witwatersrand, Johannesburg, South Africa; p Global Healthcare Consulting, Delhi, India; q ISGlobal, Barcelona Ctr. Int. Health Res. (CRESIB), Hospital Clínic – University of Barcelona, Barcelona, Spain; r Erasmus University Medical Center, Rotterdam, The Netherlands

**Keywords:** Congenital microcephaly, Adverse event, Immunisation, Guidelines, Case definition, Brighton Collaboration, GAIA

## 1 Preamble

### 1.1 Need for developing case definitions and guidelines for data collection, analysis, and presentation for congenital microcephaly as an adverse event following maternal immunisation

Congenital microcephaly, also referred to as primary microcephaly due to its presence in utero or at birth, is a descriptive term for a structural defect in which a fetus or infant’s head (cranium) circumference is smaller than expected when compared to other fetuses or infants of the same gestational age, sex and ethnic background.

Congenital microcephaly can be diagnosed either postnatally or prenatally and is usually defined by the measurement of occipital-frontal circumference (head circumference) that is more than 2 standard deviations (SDs) below the mean for age and sex or less than the 3rd percentile for age and sex [1], [2], [3]. Severe microcephaly is defined as head circumference more than 3 SDs below the mean for age and sex [4], [5], [6], [7].

Congenital microcephaly may occur as an isolated structural birth defect or in combination with other birth defects. Physiologically, congenital microcephaly is a disorder of reduced brain size and volume resulting from abnormal fetal development. Microcephaly has been associated with intellectual disability [8].

In addition to congenital microcephaly, there is also an acquired form of microcephaly in which an infant’s head circumference falls within the normal range at birth with subsequent development of microcephaly over time due to deceleration of brain growth. Classifying microcephaly as either congenital or acquired is the currently favored nomenclature rather than the past designations of “primary,” “pure,” or “true” for congenital microcephaly versus “secondary” or “syndromic” for acquired microcephaly. We have focused on congenital microcephaly for this case definition and will not address acquired microcephaly.

The term “relative microcephaly” is used when an infant is below standard weight and length for gestational age and sex with a proportionally small head circumference measurement. This constellation may be associated with a better intellectual prognosis than “absolute microcephaly” or congenital microcephaly, in which weight and length are normal for gestational age and sex [4]. Terms such as microcephaly or microencephalia are used interchangeably when referring to reduction in brain mass, rather than decreased head circumference. Thus, despite congenital microcephaly typically being associated with a small head circumference, in the case of hydrocephalus, since there can be reduced brain mass with a normal or enlarged head circumference due to enlarged ventricles from excess central nervous system fluid it would still be considered microcephaly.

A variety of estimates of the incidence of congenital microcephaly have been published in the literature, reflecting the heterogeneous definitions and methods used. Studies evaluating population level prevalence are limited as most available reports are based on small case numbers and focus on discrete populations such as individuals with cerebral palsy or musculoskeletal defects, making these studies poorly generalizable.

Incidence rates of congenital microcephaly have been estimated to vary between 0.58 and 1.87 per 10,000 live births in studies conducted in the United States and Europe [9]. While a series of 360 births with congenital microcephaly in Missouri, United States in the 1990s suggested a population incidence of more than 7 cases per 10,000 births [10], more recent data estimate congenital microcephaly rates from 2 to 12 cases per 10,000 livebirths [11].

There are very limited data on the prevalence of congenital microcephaly in low and middle-income countries (LMIC). A systematic review of 9 studies from India indicate a pooled prevalence rate of newborns with congenital microcephaly of 2.3 per 10,000 births (95% confidence interval [CI] 1.82–2.78) among 97,155 births [12]. An increase in the reported prevalence of microcephaly was noted in some areas of Brazil during 2015 where there was confirmed Zika virus transmission [13]. Prevalence of microcephaly in the 15 states of Brazil with laboratory-confirmed Zika virus transmission was 2.8 cases per 10,000 live births, which was significantly higher than in the four Brazilian states without Zika virus transmission (prevalence 0.6 cases per 10,000 live births). Another review from North Eastern Brazil employing three different criteria showed markedly varying rates [14]. In this review covering a period from 2012 to 2015, reported prevalence rates among 16,208 infants ranged from 2.1 to 8.0% based on the different criteria for congenital microcephaly used. It should be noted that in addition to microcephaly, Zika virus infection has been associated with other neurologic and brain abnormalities, which can be found in the absence of microcephaly [15], [16], [17], [18], [19], [20].

The causes of congenital microcephaly are extensive, highly variable and heterogeneous, and include both known and undetermined aetiologies. Any condition that affects the process of brain growth can result in microcephaly [21]. Table 1 is a reproduced table which provides an extensive list of genetic disorders including metabolic disorders, perinatal brain injury due to maternal disease or teratogen exposure (including in utero drug or toxin exposure and infectious agents such as toxoplasmosis, rubella, cytomegalovirus, Herpes simplex, syphilis, parvovirus B19, and varicella [TORCH infections]) during pregnancy [22]. These in utero exposures along with postnatal brain injury due to infections, infarction or trauma represent the most common known causes of microcephaly.

**Table 1:** Causes of primary *[congenital]* microcephaly: overview.

1. **Genetic causes** **Numerical chromosomal aberrations or microdeletion and/or duplication syndromes** Trisomy 13, 18, 21, etc. **Monogenetic microcephaly** Autosomal recessive microcephaly (MCPH1-10, MCPHA) Nijmegen breakage syndrome (MIM#251260) Autosomal dominant microcephaly X-chromosomal microcephaly Aicardi–Goutieres syndrome (MIM#225750, 610329, 610181, 610333, 612952) Cockayne syndrome (MIM#216400, 133540, 216411) Cornelia de Lange syndrome (MIM#122470, 610759, 614701, 300590, 300822) Rubinstein–Taybi syndrome (MIM#180849) Feingold syndrome (MIM#164280, 614326) Rett syndrome, congenital (MIM#164874) Mowat–Wilson syndrome (MIM#235730) Smith–Lemli–Opitz syndrome (MIM#270400) Seckel syndrome (MIM#210600, 606744, 608664, 613676, 613823, 61472) Ligase IV syndrome (MIM #606593) Mutations in ATRX gene (MIM*300032) Mutations in ARX gene (MIM*300382) Mutations in PQBP1 gene (MIM*300463) Mutations in ASNS gene (MIM*108370) Borjeson–Forssman–Lehmann syndrome (MIM#301900) **Imprinting disorders** Angelman syndrome (MIM#105830) 2. **Metabolic cause (genetic aetiology)** Serine biosynthesis disorder Sterol biosynthesis disorder Mitochondriopathy, e.g. pyruvate dehydrogenase deficiency Congenital disorders of glycosylation syndrome Rare congenital metabolic diseases (see text) 3. **Exogenic factors** **Intrauterine infection** Toxoplasmosis, rubella, cytomegalovirus, herpes simplex, varicella zoster virus, syphilis, human immunodeficiency virus, Zika Virus^a^, Lymphocytic Choriomeningitis Virus (LCMV)^a^ Teratogens Alcohol, cocaine, antiepileptic drugs, lead/mercury intoxication, radiation **Disruptive incident** Vascular incident (stroke), intrauterine death of twin **Maternal disease** Hyperphenylalaninaemia Maternal anorexia nervosa **Extreme insufficiency of placenta** 4. **Craniosynostosis**

Reproduced with permission from Von der Hagen et al. [22].

a Not included in original table from Von der Hagen et al.

In the largest published cohort of infants with microcephaly, genetic causes accounted for approximately one third of cases followed by perinatal brain injury and postnatal brain injury [22]. Approximately 40% of cases are of unknown aetiology. Genetic causes consist of rare inherited autosomal recessive conditions (primary autosomal recessive microcephaly) and syndromes resulting in defects in DNA repair or neuronal migration and disorders of telencephalic cleavage [23], [24]. More than 1100 clinical syndromes associated with the clinical sign, “microcephaly,” were recorded in the Online Mendelian Inheritance in Man (OMIM http://www.ncbi.nlm.nih.gov/omim) as of June 2016 [25].

Multiple studies have evaluated associations between both recommended and inadvertent vaccination in pregnancy and subsequent diagnosis of congenital anomalies in the offspring [26], [27], [28], [29], [30], [31], [32], [33], [34], [35], [36], [37], [38], [39], [40], [41], [42], [43], [44], [45], [46], [47], [48], [49], [50], [51]. While these reports do not document an association between vaccination in pregnancy and increased rates of congenital anomalies, some studies are limited by small sample sizes and lack of standardized definitions for outcomes makes it difficult to compare studies. No cases of congenital microcephaly were reported to the Vaccine Adverse Event Reporting System following maternal immunisation with the tetanus, diphtheria and acellular pertussis vaccine (Tdap) during pregnancy for the time from 2011 to 2015 [52]. The authors of the Congenital Anomalies GAIA-Brighton Case Definition reviewed studies evaluating associations between vaccination in pregnancy, including both vaccines routinely recommended during pregnancy (influenza and Tdap) and vaccinations inadvertently administered during pregnancy (live virus vaccines, Human Papillomavirus [HPV], and meningococcal vaccines), and found no increased risk of congenital anomalies, including congenital microcephaly [53].

There is no uniformly accepted definition of congenital microcephaly as an adverse event in a fetus or infant following maternal immunisation. As previously discussed, maternal immunisation has not been associated with congenital microcephaly in offspring. The goal of developing this guideline is to improve and standardize data collection and interpretation in order to evaluate for associations between maternal immunisation and congenital microcephaly. The intent of this document is to provide a standardized case definition and guidelines to improve reliability and comparability of data collected in clinical trials and observational studies and to provide a standardized framework for consistently monitoring the safety of vaccines currently recommended during pregnancy or available to women of reproductive age. The case definitions and guidelines are intended to be applicable in diverse geographic, administrative, and cultural regions, adaptable to both high and low resource settings.

### 1.2 Methods for the development of the case definition and guidelines for data collection, analysis, and presentation for congenital microcephaly as an adverse event following maternal immunisation

Following the process described in the overview paper [54] as well as on the Brighton Collaboration Website http://www.brightoncollaboration.org/internet/en/index/process.html, the Brighton Collaboration *Congenital Microcephaly Working Group* was formed in 2016 and included members with background in clinical medicine, paediatrics, neonatology, neurology, vaccinology, research, public health and industry. The composition of the working and reference group as well as results of the web-based survey completed by the reference group with subsequent discussions in the working group can be viewed at: http://www.brightoncollaboration.org/internet/en/index/working_groups.html.

To guide the decision-making for the case definition and guidelines, a literature search was performed using Medline, Embase and Scopus, including the terms (vaccin∗ and pregnan∗).ti. AND (microcephaly or microencephaly).mp. OR (immunisation∗ and pregnan∗).ti. AND (microcephaly or microencephaly).mp. OR (microcephaly or microencephaly or small head∗).sh,kw. AND (immunisation∗ and pregnan∗).sh,kw. OR ((vaccine∗ or vaccination∗) and pregnant∗).sh,kw. AND (microcephaly or microencephaly).mp. OR (maternal vaccin∗ or maternal immunisation∗ or maternal immunisation∗).mp. AND (microcephaly or microencephaly).mp. The search resulted in the identification of 23 references written in English. All abstracts were screened for possible reports of congenital microcephaly following maternal immunisation. Nine articles with potentially relevant material were reviewed in more detail, in order to identify studies using case definitions or, in their absence, providing clinical descriptions of the case material. We also reviewed additional publications related to the field. This review resulted in a detailed summary of 27 articles, including information on the study type, the vaccine, the diagnostic criteria or case definition put forth, the time interval since time of immunisation, and any other symptoms. Multiple general medical, pediatric and infectious disease textbooks were included in the search criteria.

Findings from the literature search were for the most part single case reports, in which the terminology was very inconsistent with no standard case definitions. An inventory comprising 3 relevant case definitions of congenital microcephaly was made available to working group members.

### 1.3 Rationale for selected decisions about the case definition of congenital microcephaly as an adverse event following maternal immunisation

The timing of clinical recognition of congenital microcephaly varies by setting. In some high resource settings, microcephaly may be diagnosed prenatally through ultrasound or other advanced imaging. If not detected prenatally, as is often the case in low resource settings, congenital microcephaly is most commonly diagnosed postnatally, in the first few days following birth or during autopsy of stillbirths or spontaneous or therapeutic abortions.

Thus, congenital microcephaly can be classified as diagnosed “postnatally” or “prenatally”, based exclusively on the timing of when the diagnosis of microcephaly is made [5]. We have incorporated this additional level of classification into our case definition. Within the definition context, we have assigned levels of diagnostic certainty to both postnatally and prenatally diagnosed congenital microcephaly. The diagnostic levels must not be misunderstood as reflecting different grades of clinical severity. They instead reflect diagnostic certainty (see below).

It needs to be emphasised that the grading of definition levels is used to determine diagnostic certainty, not the clinical severity of an event. Thus, a clinically very severe event may appropriately be classified as Level Two or Three rather than Level One if there is a lack of diagnostic criteria. Detailed information about the severity of the event should always be recorded, as specified by the data collection guidelines.

The number of symptoms and/or signs that will be documented for each case may vary considerably. The case definition has been formulated such that the Level One definition is highly specific for the condition. As maximum specificity normally implies a loss of sensitivity, additional diagnostic levels have been included in the definition, offering a stepwise increase of sensitivity from Level One down to Level Four or Five, while retaining an acceptable level of specificity at all levels. In this way it is hoped that all possible cases of congenital microcephaly can be captured.

As noted above, congenital microcephaly may exist alone, in the presence of other congenital anomalies, or as part of a syndrome. It is possible that congenital microcephaly may be related to or discovered after spontaneous abortion, stillbirth or an elective therapeutic abortion, and thus data collection should not be limited to live births. Thus, pathology findings of head circumference measurement performed during autopsy of stillbirth or spontaneous or therapeutic abortion are included in the case definition. We have also included radiology findings, specifically fetal ultrasound examination results, for use in the prenatal definition for congenital microcephaly.

Laboratory findings are not included in the case definition. However laboratory data (e.g., genetic test results) should be included as supportive data as many known causes of congenital microcephaly can be diagnosed through laboratory studies.

The meaning of “sudden onset” and “rapid progression” in the context of congenital microcephaly is not applicable as this condition is present at birth or at the time of fetal demise. We also do not include specific time frames for onset of symptoms following immunisation.

We postulate that a definition designed to be a suitable tool for testing causal relationships requires ascertainment of the outcome (e.g., congenital microcephaly) independent from the exposure (e.g., immunisations). Therefore, to avoid selection bias, a restrictive time interval from immunisation during pregnancy to onset of congenital microcephaly should not be an integral part of such a definition. Instead, where feasible, details of this interval should be assessed and reported as described in the data collection guidelines.

Further, congenital microcephaly often occurs outside the controlled setting of a clinical trial or hospital. In some settings it may be impossible to obtain a clear timeline of the event or diagnosis either prenatally or at birth, particularly in less developed or rural settings. In order to avoid selecting against such cases, the Brighton Collaboration case definition avoids setting arbitrary time frames.

Consistent with the Brighton Case Definition for congenital anomalies [53], we note that the first trimester of pregnancy is considered the most critical period for teratogen exposure with regards to subsequent effects on fetal development [55]. However, we believe it is important to record the time interval between maternal immunisation at any time during pregnancy and the diagnosis of congenital microcephaly in order to best evaluate the association between maternal vaccination and congenital microcephaly. Additionally, it is important to differentiate congenital microcephaly due to a known cause from congenital microcephaly without clear aetiology. Again, consistent with the Brighton Case Definition for congenital anomalies, we recommend altering the analysis plan if a study includes congenital microcephaly cases with well-known causes.

### 1.4 Guidelines for data collection, analysis and presentation

As mentioned in the overview paper, the case definition is accompanied by guidelines, which are structured according to the steps of conducting a clinical trial or observational study, i.e., data collection, analysis and presentation. Neither case definition nor guidelines are intended to guide or establish criteria for management of ill infants, children, or adults. Both were developed to improve data comparability.

### 1.5 Periodic review

Similar to all Brighton Collaboration case definitions and guidelines, review of the definition with its guidelines is planned on a regular basis (i.e., every three to five years) or more often if needed.

## 2 Case definition of congenital microcephaly^3^

### 2.1 For all levels of diagnostic certainty

Congenital Microcephaly is a clinical syndrome based on head circumference (HC) measurements. Depending on when the diagnosis is made, congenital microcephaly is stratified into the following categories:

A. Postnatally diagnosed (after birth) congenital microcephaly
B. Prenatally diagnosed (in utero) congenital microcephaly

**Fig. 1:**
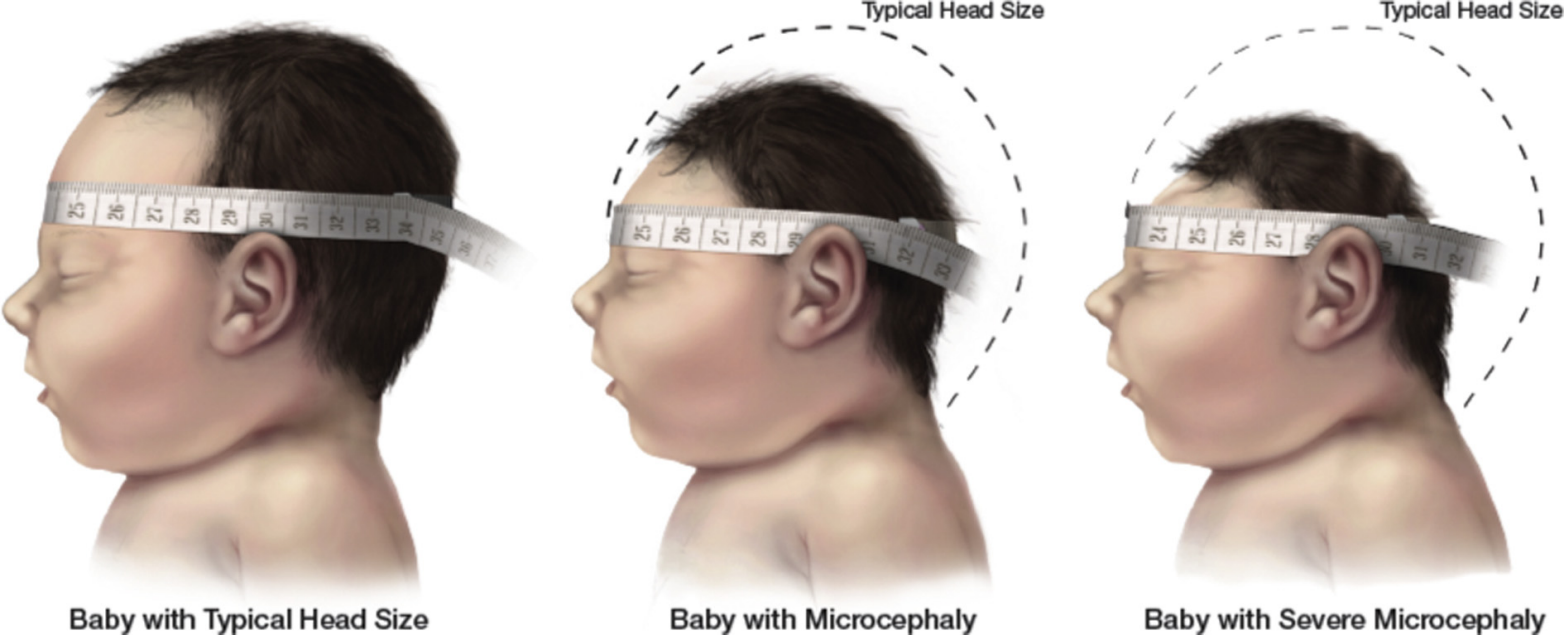
Measuring Head Circumference (image reproduced from reference CDC’s response to Zika [56]).

**Fig. 2:**
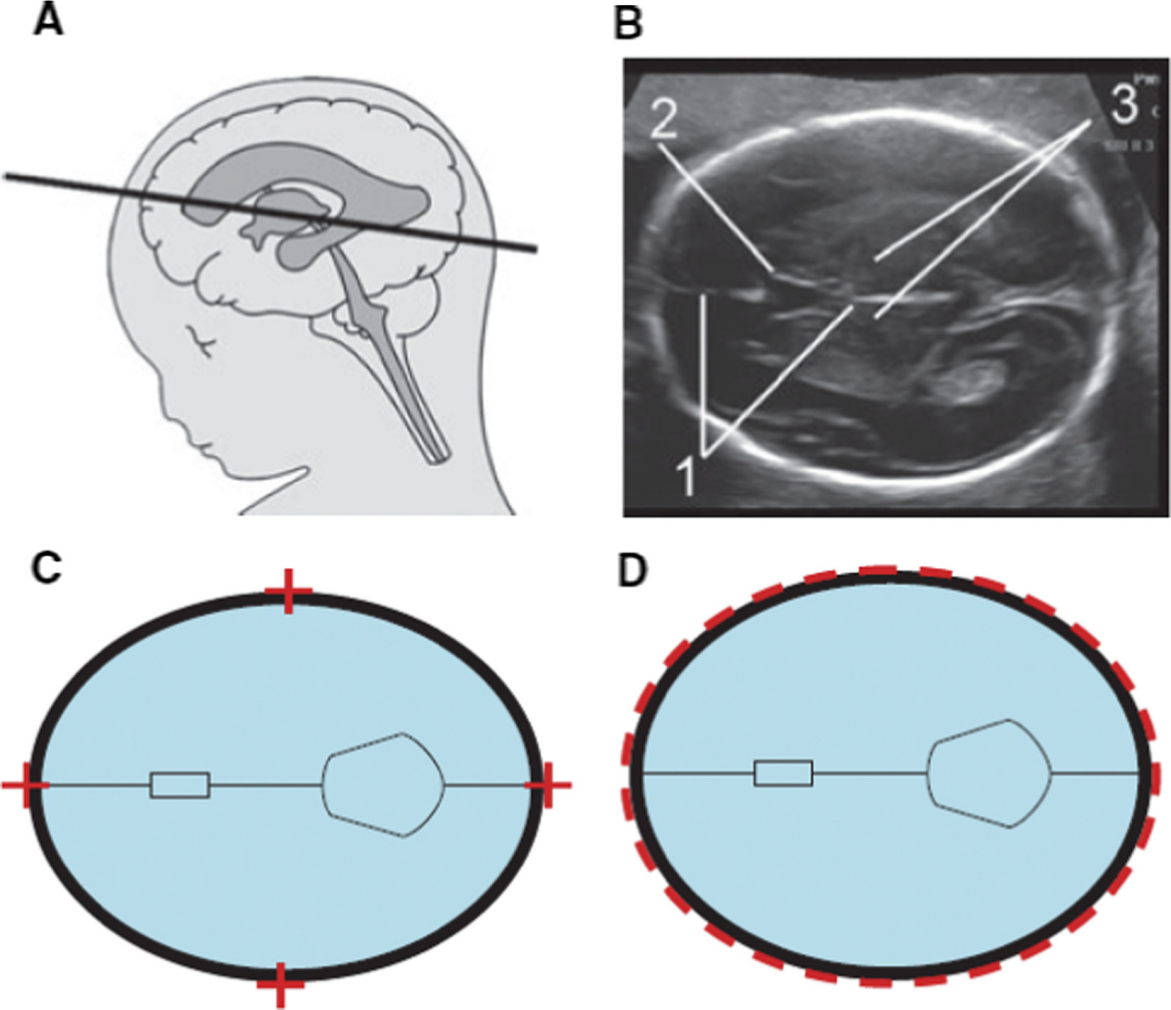
Reproduced from reference [59]. The level of the cross-section through the fetal head for correct measurement (A). The image (B) is well magnified, the head is horizontal, oval in shape and symmetrical. The landmarks are seen with a centrally positioned and continuous midline falx cerebri (1), the midline echo is broken anteriorly at one-third of its length by the cavum septi pellucidi (2) and the thalami are located symmetrically (3). Callipers, are placed so that their intersection is on the outer border of the bones (C). When using the ellipse facility this should run along the outer border of the skull (D).

In order to apply the case definition of postnatally diagnosed congenital microcephaly, it is necessary to first obtain an accurate HC measurement using a flexible, non-stretchable measuring tape. We recommend using a disposable paper tape measure or a plastic tape measure in which one end inserts into the other. The use of a metal tape measure is discouraged due to the risk of inadvertent laceration of the newborn’s skin. Whichever tape measure is used, the metric system should be used and marked by 0.1 cm increments. To measure the HC, securely wrap the tape measure around the widest possible circumference of the infant’s head (typically, 1–2 finger-widths above the eyebrow (supraorbital ridges) on the forehead, above the ears, to the most prominent part of the back of the head (occiput) (Fig. 1). Please note that the occiput may not always be easily recognizable, particularly if there is significant molding of the infant’s head. Therefore, it is important to repeat the measurement three times and to select the largest measurement to the nearest 0.1 cm [56] (see Fig. 2).

Head Circumference Tape Measure Checklist:

• Flexible, non-stretchable, plastic or disposable paper
• 1–2 cm (1/4–1/2 in.) wide
• Marked in 0.1 cm increments

Utilization of appropriate HC reference charts is recommended (see Appendix A) such as WHO Child Growth Standards [57] and Intergrowth 21st charts [58]. It is recommended to record the actual measurement of the head circumference in addition to percentile.

In order to apply the case definition of prenatally diagnosed congenital microcephaly, it is necessary to obtain an accurate HC measurements via prenatal ultrasound (US) scan by a sonographer or a health professional trained in sonography. A fetal HC measurement can be obtained starting at approximately 14 weeks estimated gestational age. Using the US machine’s ellipse facility, the lines of the ellipse should be placed on the outer border of the fetal skull in order to obtain the HC measurement [59].

General recommendations for optimal HC measurement in a fetus with normal intracranial anatomy include [59]:

- Obtaining a cross-sectional view of the fetal head at the level of the thalami.
- The cross-section view of the fetal head should take up at least 30% of the ultrasound monitor.
- Ensure that the skull is oval, symmetrical and visible all the way around on the ultrasound monitor.
- Intracranial anatomy should be visible with centrally positioned continuous medline echo (falx cerebri) broken anteriorly by the cavum septum pellucidum and with the thalami visible symmetrically on each side of the midline.

Accurate gestational age determination is vital when determining congenital microcephaly based on prenatal ultrasound scan. Ideally, dating is based on or confirmed by a first trimester ultrasound scan using crown-rump length for measurement. If after the first trimester, gestational age has not yet been confirmed and congenital microcephaly is suspected, HC should not be used to determine gestational age [60]. In cases of congenital microcephaly, intracranial anatomy may be distorted and other associated intracranial findings may be present [61].

There are currently several fetal growth standards in use for head circumference measurements including those from the Fetal Growth Longitudinal Study of the INTERGROWTH-21st Project, the WHO Multicentre Growth Reference Study (MGRS), the National Institute of Child Health and Human Development (NICHD) Fetal Growth Studies, and those referenced by the United States Society for Maternal-Fetal Medicine (SMFM) based on Hadlock growth curves [61], [62], [63], [64]. The head circumference measurements of the Intergrowth 21st Project with its international standards is the most applicable to multinational vaccine trials. The standards are very similar to those of the WHO MGRS, are readily available online and are endorsed by the International Society for Ultrasonography in Obstetrics and Gynecology [60], [63]. Given the prevalence of Hadlock growth measurements in ultrasonography software, using the head circumference guidelines from SMFM may also be acceptable for prenatally diagnosed congenital microcephaly.

It should be noted that while all levels may be used in any setting, it is most likely that level 1 and 2 definitions for postnatally diagnosed congenital microcephaly will be primarily used in high resource settings with access to prenatal ultrasound. Because prenatally diagnosed congenital microcephaly relies on the use of ultrasound technology, it is not feasible to diagnose this condition in areas without access to ultrasound machines.

**Table:** 

***Definitions of terms used***[65]:	
● **Intrauterine Insemination (IUI)** – A procedure in which a fine catheter is inserted through the cervix into the uterus to deposit a sperm sample directly into the uterus, to achieve fertilization and pregnancy.	
● **Embryo Transfer** – the procedure in which one or more embryos are placed in the uterus or fallopian tube.	
● **Ultrasound (US)^62^**	- 1st trimester (⩽13 6/7 weeks)
	- 2nd trimester scan (14 0/7–27 6/7 weeks)
	- 3rd trimester (28 0/7 + weeks)
● **Last Menstrual Period (LMP)** – Gestational age is calculated from the first day of the mother’s last menstrual period**.**	

**A. Postnatally diagnosed Congenital Microcephaly Case Definition**

Level 1 of diagnostic certainty

1. Live birth, stillbirth, or spontaneous or therapeutic abortion of at least 24 weeks of Gestational Age (GA)∼
  AND
2. HC 2 SD below mean or <3 percentile according to GA and gender, using appropriate standardized reference charts for the population (e.g., WHO growth reference charts if GA ⩾37 weeks and Intergrowth-21st reference charts for GA 24–36 weeks)
  AND
3. Measured between 24 and 36 h after birth or end of pregnancy.
∼GA assessed based on certain LMP with confirmatory 1st trimester or 2nd trimester US scan, IUI, or embryo transfer date

Level 2A of diagnostic certainty

1. Live birth, stillbirth, or spontaneous or therapeutic abortion of at least 24 weeks of GA∼
  AND
2. HC 2 SD below mean or <3 percentile according to GA and gender, using appropriate standardized reference charts for the population (e.g., WHO growth reference charts if GA ⩾37 weeks and Intergrowth-21st reference charts for GA 24–36 weeks)
  AND
3. Measured within the first 24 h §
  OR
  Measured >36 h and up to 6 weeks after birth or end of pregnancy with no apparent post-natal insult resulting in microcephaly
  ∼GA assessed based on certain LMP with confirmatory 1st trimester or 2nd trimester US scan, IUI, or embryo transfer date

Level 2B of diagnostic certainty

1. Live birth, stillbirth, or spontaneous or therapeutic abortion of at least 24 weeks of GA∼
  AND
2. HC 2 SD below mean or <3 percentile according to GA and gender, using appropriate standardized reference charts for the population (e.g., WHO growth reference charts if GA ⩾37 weeks and Intergrowth-21st reference charts for GA 24–36 weeks)
  AND
3. Measured within the first 24 h §
  OR
  Measured >36 h and up to 6 weeks after birth or end of pregnancy with no apparent post-natal insult resulting in microcephaly
  ∼GA assessed based on uncertain LMP with 2nd trimester US scan
  §Take into account the variability in this period based on molding of the head

Level 3A of diagnostic certainty

1. Live birth, stillbirth, or spontaneous or therapeutic abortion of at least 24 weeks of GA∼
  AND
2. HC 2 SD below mean or <3 percentile according to GA and gender, using appropriate standardized reference charts for the population (e.g., WHO growth reference charts if GA ⩾37 weeks and Intergrowth-21st reference charts for GA 24–36 weeks)
  AND
3. Measured up to 6 weeks after birth or end of pregnancy with no apparent post-natal insult resulting in microcephaly
∼GA based on LMP without confirmatory 1st or 2nd trimester ultrasound

Level 3B of diagnostic certainty

1. Live birth, stillbirth, or spontaneous or therapeutic abortion
  AND
2. Case meets criteria for microcephaly using a validated algorithm: 1 inpatient diagnosis OR 2 outpatient diagnoses OR 1 outpatient diagnosis AND death in first year using the following diagnostic codes ICD-9-CM code 742.1 or ICD-10-CM code Q02

Level 4 of diagnostic certainty

1. Live birth, stillbirth, or spontaneous or therapeutic abortion
  AND
2. Diagnosis of congenital microcephaly based on physical inspection without HC measurement
  OR
  Diagnosis of congenital microcephaly based on ICD-9-CM or ICD-10-CM code that does not meet validated algorithm criteria above.

**B. Prenatally diagnosed Congenital Microcephaly Case Definition**

Level 1A of diagnostic certainty

1. Fetus of at least 24 weeks GA based on certain LMP with confirmatory 1st trimester or 2nd trimester US scan IUI, or embryo transfer date
  AND
2. HC 2 SD below mean or <3 percentile according to fetal US scan using appropriate standardized reference charts according to GA and gender for the population (e.g., WHO growth reference charts if GA ⩾37 weeks and Intergrowth-21st reference charts for GA 24–36 weeks)
  AND
3. Confirmation of microcephaly (i.e., HC 2 SD below mean or <3 percentile) in fetus by at least one additional US scan after 24 weeks and at least one week after first US
  OR
  Confirmation of microcephaly by HC measurement with standard tape measure at birth or autopsy

Level 1B of diagnostic certainty

1. Fetus of at least 24 weeks GA based on uncertain LMP with 2nd trimester US
  AND
2. HC 2 SD below mean or <3 percentile according to fetal ultrasound (US) examination using appropriate standardized reference charts according to GA and gender for the population (e.g., WHO growth reference charts if GA ⩾37 weeks and Intergrowth-21st reference charts for GA 24–36 weeks)
  AND
3. Confirmation of microcephaly (i.e., HC 2 SD below mean or <3 percentile) in fetus by at least one additional US scan after 24 weeks and at least one week after first US
  OR
  Confirmation of microcephaly by HC measurement with standard tape measure at birth or autopsy

Level 2 of diagnostic certainty

1. Fetus of at least 24 weeks GA based on certain or uncertain LMP with fundal height and no confirmatory 1st or 2nd trimester US scan
  AND
2. HC 2 SD below mean or <3 percentile according to fetal US scan using appropriate standardized reference charts according to GA and gender for the population (e.g., WHO growth reference charts if GA ⩾37 weeks and Intergrowth-21st reference charts for GA 24–36 weeks) with femur length and abdominal circumference concordant with GA assessment
  AND
3. Confirmation of microcephaly (i.e., HC 2 SD below mean or <3 percentile) in fetus with at least one additional US scan after 24 weeks and at least one week after first US
  OR
  Confirmation of microcephaly by HC measurement with standard tape measure at birth or autopsy

Level 3A of diagnostic certainty

1. Fetus of at least 24 weeks GA based on certain LMP with confirmatory 1st trimester or 2nd trimester US scan, uncertain LMP with 2nd trimester US, IUI, or embryo transfer date
  AND
2. HC 2 SD below mean or <3 percentile according to fetal US scan using appropriate standardized reference charts according to GA and gender for the population (e.g., WHO growth reference charts if GA ⩾37 weeks and Intergrowth-21st reference charts for GA 24–36 weeks) with femur length and abdominal circumference concordant with GA assessment
  AND
3. No additional data to confirm microcephaly (i.e., No additional prenatal US scan or confirmation of microcephaly by HC measurement at birth or autopsy)

Level 3B of diagnostic certainty

1. Fetus of at least 24 weeks GA based on certain or uncertain LMP with fundal height and no confirmatory 1st or 2nd trimester US scan
  AND
2. HC 2 SD below mean or <3 percentile according to fetal US scan using appropriate standardized reference charts according to GA and gender for the population (e.g., WHO growth reference charts if GA ⩾37 weeks and Intergrowth-21st reference charts for GA 24–36 weeks) with femur length and abdominal circumference concordant with GA assessment
  AND
3. No additional data to confirm microcephaly (i.e., No additional prenatal US scan or confirmation of microcephaly by HC measurement at birth or autopsy)

Level 4 of diagnostic certainty

1. Fetus of at least 24 weeks GA based on certain LMP with confirmatory 1st trimester or 2nd trimester US scan, uncertain LMP with 2nd trimester US, IUI, embryo transfer date, or certain or uncertain LMP with fundal height and no confirmatory 1st or 2nd trimester US scan
  AND
2. HC 2 SD below mean or <3 percentile according to fetal US scan using appropriate standardized reference charts according to GA and gender for the population (e.g., WHO growth reference charts if GA ⩾37 weeks and Intergrowth-21st reference charts for GA 24–36 weeks)
  AND
3. HC at birth or autopsy is in the normal range using appropriate standardized reference charts according to GA and gender for the population, which means that this is NOT a case of prenatally diagnosed congenital microcephaly

## 3 Guidelines for data collection, analysis and presentation of congenital microcephaly

It was the consensus of the Brighton Collaboration Congenital Microcephaly Working Group to recommend the following guidelines to enable meaningful and standardized collection, analysis, and presentation of information about congenital microcephaly. However, implementation of all guidelines might not be possible in all settings. The availability of information may vary depending upon resources, geographical region, and whether the source of information is a prospective clinical trial, a post-marketing surveillance or epidemiological study, or an individual report of congenital microcephaly. Also, as explained in more detail in the overview paper in this volume, these guidelines have been developed for guidance only by this working group and are not to be considered a mandatory requirement for data collection, analysis, or presentation.

### 3.1 Data collection

These guidelines represent a desirable standard for the collection of data on availability following maternal immunisation to allow for comparability of data, and are recommended as an addition to data collected for the specific study question and setting. The guidelines are not intended to guide the primary reporting of congenital microcephaly to a surveillance system or study monitor. Investigators developing a data collection tool based on these data collection guidelines also need to refer to the criteria in the case definition, which are not repeated in these guidelines.

Guidelines 1–42 below have been developed to address data elements for the collection of adverse event information as specified in general drug safety guidelines by the International Conference on Harmonization of Technical Requirements for Registration of Pharmaceuticals for Human Use [66], and the form for reporting of drug adverse events by the Council for International Organizations of Medical Sciences [67]. These data elements include an identifiable reporter and patient, one or more prior immunisations, and a detailed description of the adverse event, in this case, of congenital microcephaly following maternal immunisation. The additional guidelines have been developed as guidance for the collection of information to allow for a more comprehensive understanding of congenital microcephaly following maternal immunisation. Furthermore, these guidelines are also called to serve as a standard guidance in the case definition of congenital microcephaly in the context of observational studies conducted in pregnant women.

#### 3.1.1 Source of information/reporter

For all cases and/or all study participants, as appropriate, the following information should be recorded:

(1) Date of report.
(2) Name and contact information of person reporting^4^ and/or diagnosing congenital microcephaly as specified by country-specific data protection law.
(3) Name and contact information of the investigator responsible for the subject, as applicable.
(4) Relation to the patient (e.g., immunizer [clinician, nurse], family member [indicate relationship], other).

#### 3.1.2 Vaccinee/control

##### 3.1.2.1 Demographics

For all cases and/or all study participants, as appropriate, the following information should be recorded:

(5) Case/study participant identifiers (e.g., medical record number) or code (or in accordance with country-specific data protection laws).
(6) Date of birth, gestional age at time of stillbirth, or gestational age at time of spontaneous or therapeutic abortion, if applicable estimated gestational age at time of fetal demise, age of mother, age of infant or gestational age of fetus, race and ethnicity of both infant and mother, and sex of fetus.
(7) For infants: Gestational age and birth weight, birth length and head circumference.

##### 3.1.2.2 Clinical and immunisation history

For all cases and/or all study participants, as appropriate, the following information should be recorded:

(8) For the mother, pre-conception medical history, including hospitalisations, underlying diseases/disorders, and medications as well as medical history during pregnancy such as exposure to substances related to congenital microcephaly, tobacco use, alcohol use, illicit drug use, pre-immunisation signs and symptoms including identification of indicators for, or the absence of, a history of allergy to vaccines, vaccine components or medications. Specific focus should be on maternal medical conditions associated with increased risk for having an infant with congenital microcephaly (e.g., anorexia nervosa).
(9) Also, for the mother, any medication history (other than treatment for the event described) prior to, during, and after immunisation including prescription and non-prescription medication a specific focus on potentially teratogenic medication exposures. Use of prenatal vitamins and folic acid should also be noted.
(10) Maternal immunisation history (i.e., previous immunisations and any adverse event following immunisation (AEFI)), in particular occurrence of congenital microcephaly in a prior pregnancy following previous maternal immunisation.

#### 3.1.3 Details of the immunisation

For all cases and/or all study participants, as appropriate, the following information should be recorded:

(11) Date and time of maternal immunisation(s).
(12) Description of vaccine(s) (name of vaccine, manufacturer, lot number, dose [e.g., 0.25 mL, 0.5 mL, etc.], and expiration date) and number of dose if part of a series of immunisations against the same disease). The composition and volume of the diluent used as well as information about whether the diluent was from the same or a separate container should also be recorded (lot number recorded if separate container).
(13) The anatomical sites (including left or right side) of all immunisations (e.g., vaccine A in proximal left lateral thigh, vaccine B in left deltoid).
(14) Route and method of administration (e.g., intramuscular, intradermal, subcutaneous, and needle-free (including type and size), other injection devices).
(15) Needle length and gauge.

#### 3.1.4 The adverse event

For all cases at any level of diagnostic certainty and for reported events with insufficient evidence, the criteria fulfilled to meet the case definition should be recorded.

Specifically document:

(16) Clinical description of signs and symptoms of congenital microcephaly, and if there was medical confirmation of the event (i.e., patient seen by physician).
(17) Date/time of first observation^5^ of congenital microcephaly and diagnostic confirmation,^6^ and final outcome.^7^
(18) Concurrent signs, symptoms, and diseases.
(19) Measurement/testing
  • Values and units of routinely measured parameters (e.g., cm, inches);
  • Method of measurement (e.g., type of measuring tape, method of measurement, etc.);
  • Results of laboratory examinations (e.g., congenital syphilis, toxoplasmosis, Zika virus infection, other congenital infections) including genetic testing, surgical and/or pathological findings and diagnoses if present (e.g., results of amniocentesis).
(20) Treatment, if any given for congenital microcephaly and any associated conditions.
(21) Physical and developmental outcome^8^ at last observation for living infants.
(22) Objective clinical evidence supporting classification of the event as “serious”.^9^
(23) Exposures other than maternal immunisation during pregnancy (e.g., maternal medications, infections, environmental) considered potentially relevant to the reported event.

#### 3.1.5 Miscellaneous/general

(24) Based on the case definition, we recommend the duration of surveillance for congenital microcephaly should begin no earlier than 24 weeks duration and extend no longer than 1 year of age, the age cut-off for microcephaly diagnosis based on diagnostic coding algorithms.
(25) The duration of follow-up reported during the surveillance period should be predefined likewise. Congenital microcephaly should be diagnosed either prenatally or during the first 6 weeks of life. Diagnoses after this time may represent acquired microcephaly rather than congenital microcephaly.
(26) Methods of data collection should be consistent within and between study groups, if applicable.
(27) Follow-up of cases should attempt to verify and complete the information collected as outlined in data collection guidelines 1–23.
(28) Investigators of patients with congenital microcephaly should provide guidance to reporters to optimise the quality and completeness of information provided.
(29) Reports of congenital microcephaly should be collected throughout the study period regardless of the time elapsed between immunisation and the adverse event. If this is not feasible due to the study design, the study periods during which safety data are being collected should be clearly defined.

### 3.2 Data analysis

The following guidelines represent a desirable standard for analysis of data on congenital microcephaly to allow for comparability of data, and are recommended as an addition to data analysed for the specific study question and setting.

(30) Reported events should be classified in one of the following five categories including the four (postnatally diagnosed congenital microcephaly) or five (prenatally diagnosed microcephaly) levels of diagnostic certainty. Events that meet the case definition should be classified according to the levels of diagnostic certainty as specified in the case definition. Events that do not meet the case definition should be classified in the additional categories for analysis.

**Event classification in 5 categories**^10^

**Event meets case definition**

(1) Level 1: *Criteria as specified in the congenital microcephaly case definition*
*Specify postnatally or prenatally diagnosed congenital microcephaly*
(2) Level 2: *Criteria as specified in the congenital microcephaly case definition*
*Specify postnatally or prenatally diagnosed congenital microcephaly*
(3) Level 3: *Criteria as specified in the congenital microcephaly case definition*
*Specify postnatally or prenatally diagnosed congenital microcephaly*

**Event does not meet case definition**

***Additional categories for analysis***

(4) Reported congenital microcephaly with insufficient evidence to meet the case definition^9^
(5) Not a case of congenital microcephaly^11^

In addition, congenital microcephaly attributed to an alternative cause (e.g., congenital CMV) should still be recorded and identified as likely attributable to a known cause.

(31) The interval between immunisation and reported congenital microcephaly could be defined as the date/time of immunisation (with regards to gestational age) to the date/time of clinical recognition^12^ of the first signs consistent with the definition.
(32) If more than one measurement of a particular criterion is taken and recorded, the value corresponding to the greatest magnitude of the adverse experience could be used as the basis for analysis. Analysis may also include other characteristics like qualitative patterns of criteria defining the event.
(33) The distribution of data (as numerator and denominator data) could be analysed in predefined increments (e.g., measured values, times), where applicable. Increments specified above should be used. When only a small number of cases is presented, the respective values or time course can be presented individually.
(34) Data on congenital microcephaly obtained from subjects receiving a vaccine should be compared with those obtained from an appropriately selected and documented control group(s) to assess background rates in non-exposed populations, and should be analysed by study arm and dose where possible, e.g., in prospective clinical trials.

### 3.3 Data presentation

These guidelines represent a desirable standard for the presentation and publication of data on congenital microcephaly following maternal immunisation to allow for comparability of data, and are recommended as an addition to data presented for the specific study question and setting. Additionally, it is recommended to refer to existing general guidelines for the presentation and publication of randomised controlled trials, systematic reviews, and meta-analyses of observational studies in epidemiology (e.g. statements of Consolidated Standards of Reporting Trials (CONSORT), of Improving the quality of reports of meta-analyses of randomised controlled trials (QUORUM), and of meta-analysis Of Observational Studies in Epidemiology (MOOSE), respectively).

(35) All reported events of congenital microcephaly should be presented according to the categories listed in guideline 30.
(36) Data on possible congenital microcephaly events should be presented in accordance with data collection guidelines 1–23 and data analysis guidelines 30–34.
(37) Terms to describe congenital microcephaly such as “low-grade”, “mild”, “moderate”, “high”, “severe” or “significant” are highly subjective, prone to wide interpretation, and should be avoided, unless clearly defined.
(38) Data should be presented with numerator and denominator (n/N) (and not only in percentages), if available.

Although immunisation safety surveillance systems denominator data are usually not readily available, attempts should be made to identify approximate denominators. The source of the denominator data should be reported and calculations of estimates be described (e.g., manufacturer data like total doses distributed, reporting through Ministry of Health, coverage/population based data, etc.).

(39) The incidence of cases in the study population should be presented and clearly identified as such in the text.
(40) If the distribution of data is skewed, median and range are usually the more appropriate statistical descriptors than a mean. However, the mean and standard deviation should also be provided.
(41) Any publication of data on congenital microcephaly should include a detailed description of the methods used for data collection and analysis as possible. It is essential to specify:
  • The study design;
  • The method, frequency and duration of monitoring for congenital microcephaly;
  • The trial profile, indicating participant flow during a study including drop-outs and withdrawals to indicate the size and nature of the respective groups under investigation;
  • The type of surveillance (e.g., passive or active surveillance);
  • The characteristics of the surveillance system (e.g., population served, mode of report solicitation);
  • The search strategy in surveillance databases;
  • Comparison group(s), if used for analysis;
  • The instrument of data collection (e.g., standardized questionnaire, diary card, report form);
  • Whether the day of immunisation was considered “day one” or “day zero” in the analysis;
  • Whether the date of onset^4^ and/or the date of first observation^4^ and/or the date of diagnosis 5 was used for analysis; and
  • Use of this case definition for congenital microcephaly, in the abstract or methods section of a publication.^13^

## 4 Disclaimer

The findings, opinions and assertions contained in this consensus document are those of the individual scientific professional members of the working group. They do not necessarily represent the official positions of each participant’s organisation (e.g., government, university, or corporation). Specifically, the findings and conclusions in this paper are those of the authors and do not necessarily represent the views of their respective institutions.

## Footnotes

3 The case definition should be applied when there is no clear alternative diagnosis for the reported event to account for the combination of symptoms.

4 If the reporting centre is different from the vaccinating centre, appropriate and timely communication of the adverse event should occur.

5 The date and/or time of first observation of the first sign or symptom indicative for congenital microcephaly can be used if date/time of onset is not known.

6 The date of diagnosis of an episode is the day post immunisation when the event met the case definition at any level.

7 E.g., recovery to pre-immunisation health status, spontaneous resolution, therapeutic intervention, persistence of the event, sequelae, death.

8 To determine the appropriate category, the user should first establish, whether a reported event meets the criteria for the lowest applicable level of diagnostic certainty, e.g., Level three. If the lowest applicable level of diagnostic certainty of the definition is met, and there is evidence that the criteria of the next higher level of diagnostic certainty are met, the event should be classified in the next category. This approach should be continued until the highest level of diagnostic certainty for a given event could be determined. Major criteria can be used to satisfy the requirement of minor criteria. If the lowest level of the case definition is not met, it should be ruled out that any of the higher levels of diagnostic certainty are met and the event should be classified in additional categories four or five.

9 An AEFI is defined as serious by international standards if it meets one or more of the following criteria: (1) it results in death, (2) is life-threatening, (3) it requires inpatient hospitalisation or results in prolongation of existing hospitalisation, (4) results in persistent or significant disability/incapacity, (5) is a congenital anomaly/birth defect, (6) is a medically important event or reaction.

10 If the evidence available for an event is insufficient because information is missing, such an event should be categorised as “Reported congenital microcephaly with insufficient evidence to meet the case definition”.

11 An event does not meet the case definition if investigation reveals a negative finding of a necessary criterion (necessary condition) for diagnosis. Such an event should be rejected and classified as “Not a case of congenital microcephaly”.

12 The date and/or time of onset is defined as the time post immunisation, when the first sign or symptom indicative for congenital microcephaly occurred. This may only be possible to determine in retrospect.

13 Use of this document should preferably be referenced by referring to the respective link on the Brighton Collaboration website (http://www.brightoncollaboration.org).
