# Supplementary material for: Congenital microcephaly: Case definition & guidelines for data collection, analysis, and presentation of safety data after maternal immunisation
Source: Vaccine. 2017 Dec 4;35(48Part A):6472–82. doi: 10.1016/j.vaccine.2017.01.044 (PMC5710988; doi:10.1016/j.vaccine.2017.01.044)

# APPENDIX A: Reference charts for head circumference

A1. [WHO Growth standards reference charts](http://www.who.int/childgrowth/standards/hc_for_age/en/) for TERM INFANTS (≥ 37 weeks of gestational age)


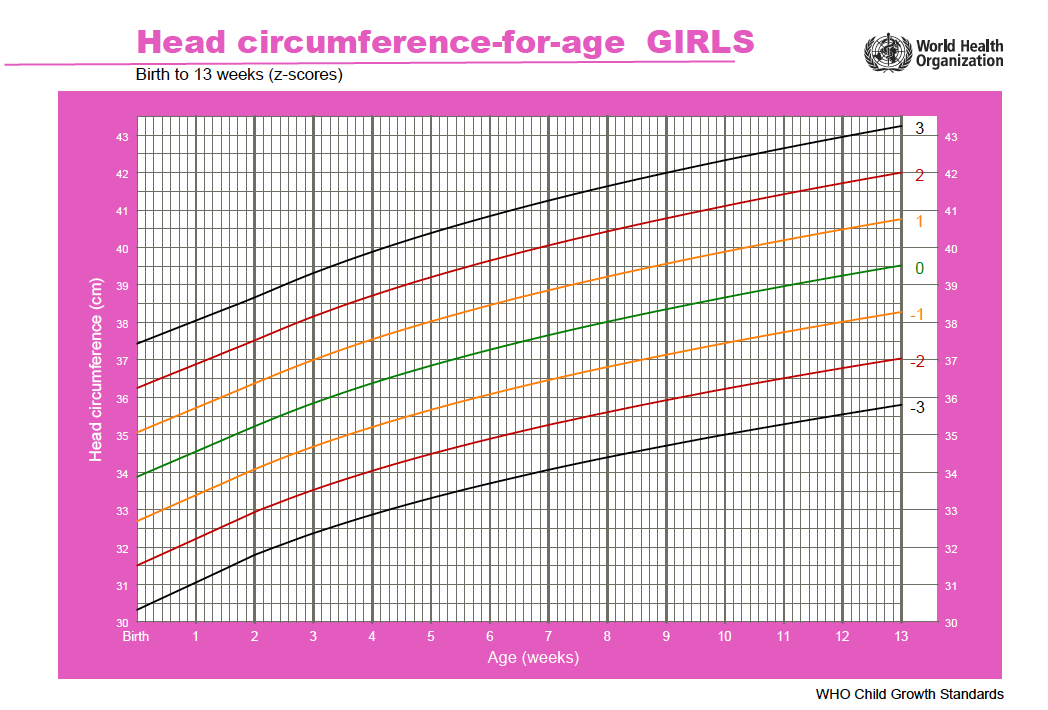


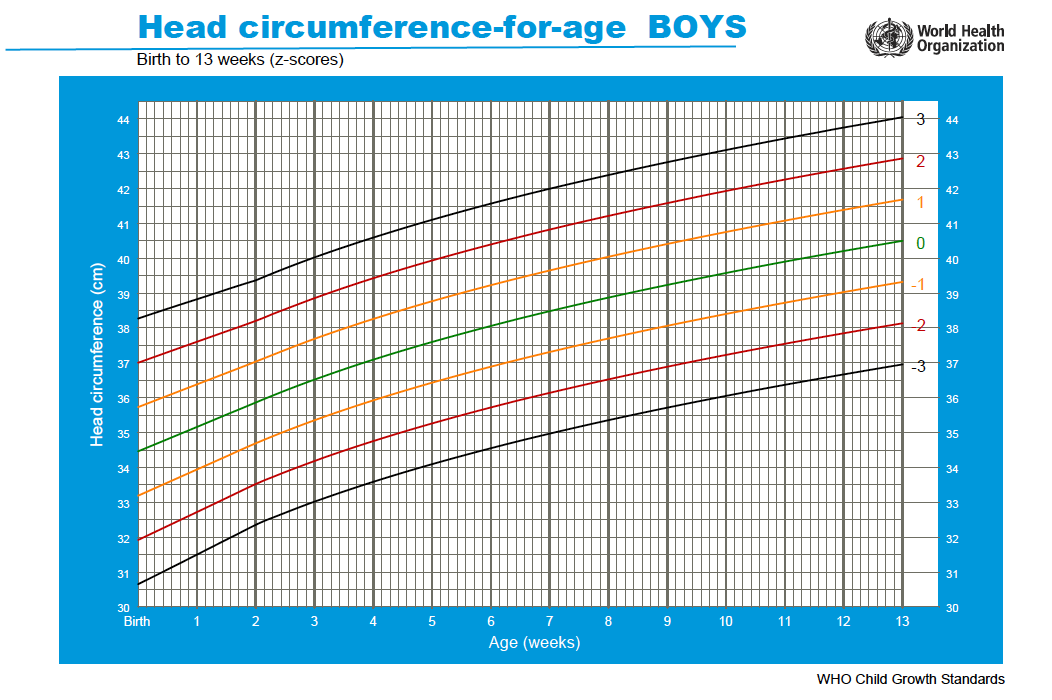


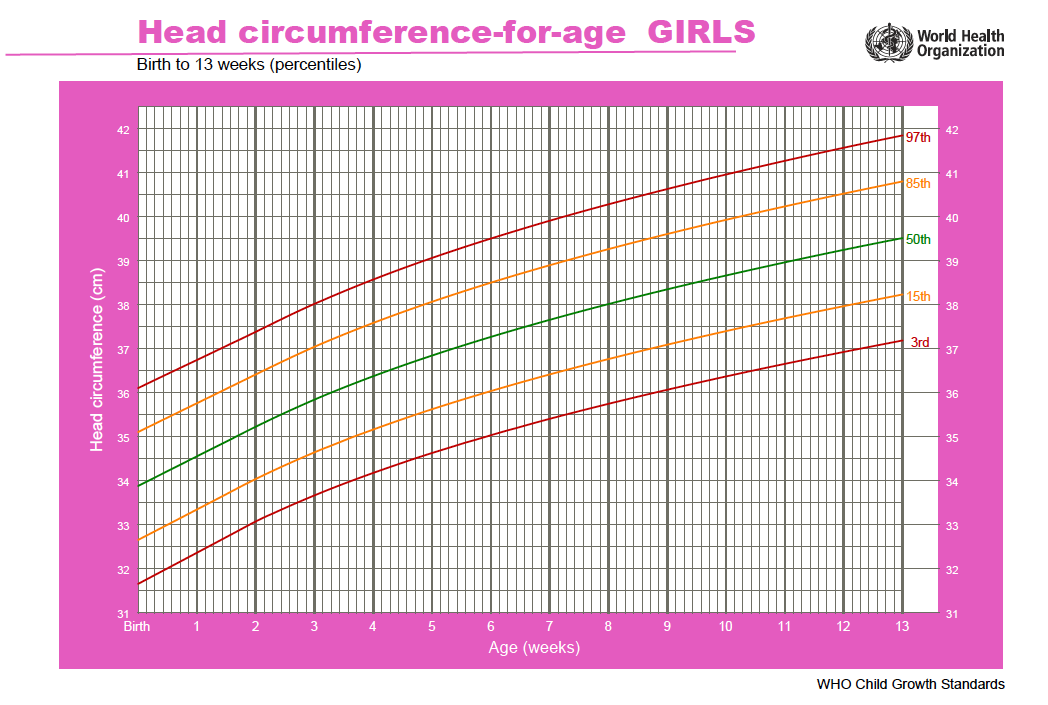


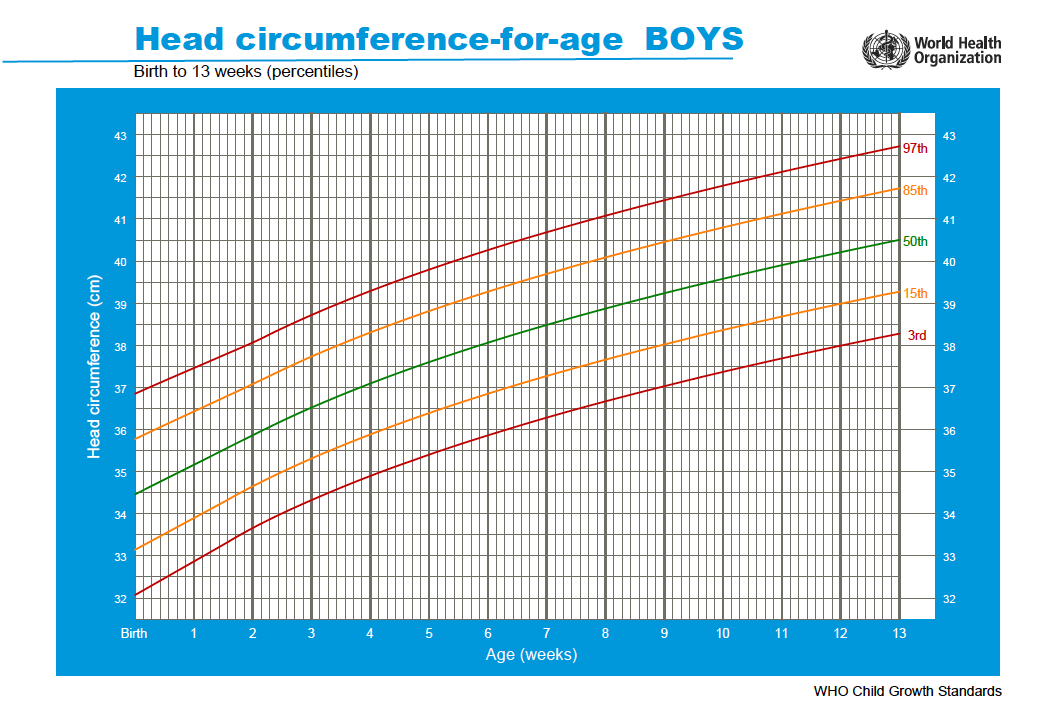


A2. [Intergrowth-21^st^ Project standards reference charts](https://intergrowth21.tghn.org/articles/new-intergrowth-21st-international-postnatal-growth-standards-charts-available/) for PRE-TERM NEONATES (24 to 36 weeks of gestational age)


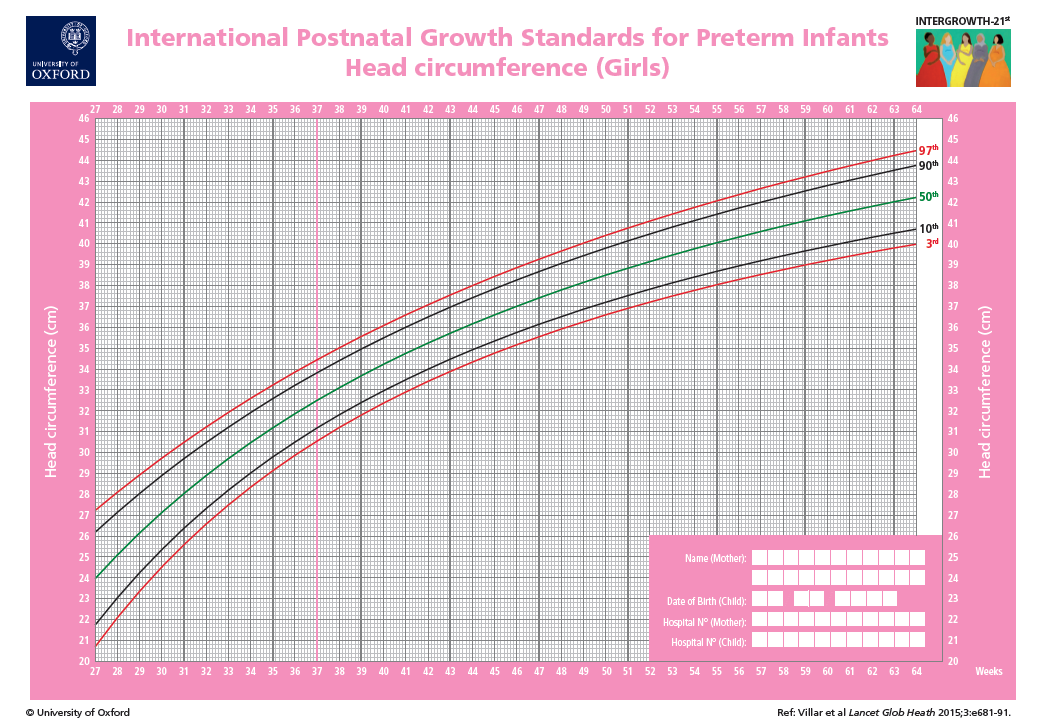


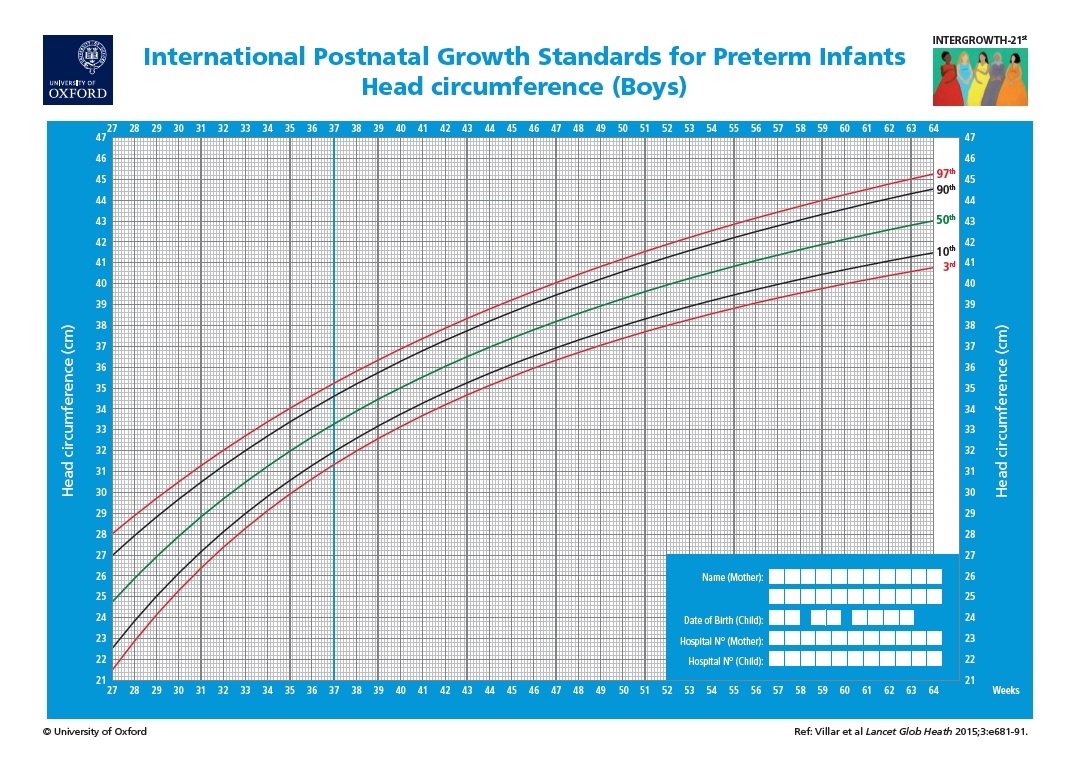

Supplement: Supplementary data 1 [file mmc1.docx]
